# Incidental vocabulary acquisition from listening to English teacher education lectures: A case study from Macau higher education

**DOI:** 10.3389/fpsyg.2022.993445

**Published:** 2022-09-02

**Authors:** Barry Lee Reynolds, Xiaowen (Serina) Xie, Quy Huynh Phu Pham

**Affiliations:** ^1^Faculty of Education, University of Macau, Taipa, Macao SAR, China; ^2^Centre for Cognitive and Brain Sciences, University of Macau, Taipa, Macao SAR, China; ^3^Moray House School of Education and Sport, The University of Edinburgh, Edinburgh, United Kingdom; ^4^Creative Language Center, Ton Duc Thang University, Ho Chi Minh City, Vietnam

**Keywords:** incidental vocabulary acquisition, lexical coverage, EMI, listening to lectures, Macau

## Abstract

Some proponents of higher education English as a medium of instruction (EMI) have suggested listening to English lectures provides students the opportunity to incidentally acquire unknown words. A case study was designed to examine this assumption. First, the lexical profiles of 27 *Introduction to English Language Teaching* first-year undergraduate course lectures were computed to determine how many words students need to know for comprehension. Then an incoming year-1 undergraduate student with an English vocabulary size of 7,500 word families and mastery of the most frequent 3,000 word families listened to these lectures across 13.5 weeks with the purpose of measuring incidental acquisition gains of three aspects of word knowledge for ten targeted words. Lastly, the student’s perceptions about listening to EMI lectures and potentials for this listening inducing incidental acquisition of word knowledge were gathered through a semi-structured interview. The lexical profiling of the entire corpus showed students need knowledge of the most frequent 4,000 English word families plus proper nouns and marginal words for 98% lexical coverage; however, some lectures present students with a more substantial lexical burden than the lectures overall. The student made the most gains in receptive meaning, followed by receptive form, and finally productive meaning. Content analysis of the interview transcript found seven themes representing the student’s perception about listening to EMI lectures and their potential for inducing incidental vocabulary acquisition. While the student found listening to the EMI lectures challenging, he perceived the process as useful in preparing for university studies and a career as a secondary English teacher. The student perceived attention, topic, existing vocabulary knowledge, lecturer’s native language, and lack of interaction with the lecturer to have moderated incidental learning of vocabulary through listening to English lectures. These results indicate a need to confirm whether incoming students’ vocabulary knowledge meet the lexical demands of the EMI lectures given in the Macau context. Furthermore, pedagogical training on teacher talk strategies and orientation training for incoming students should both be provided to ensure students are receiving high quality instruction.

## Introduction

Extant research on the incidental acquisition of second language vocabulary has mostly focused on the learning of vocabulary that occurs through reading (Reynolds and Wible, 2014). This is because the majority of exposure that second or foreign language learners have to a target language has traditionally been from reading (Nation, 2013a). However, the incidental acquisition of vocabulary from listening has begun to gain traction (e.g., van Zeeland and Schmitt, 2013; Jin and Webb, 2020). This has come along with language learners’ increased access to media through the internet and mobile networks (Soyoof et al., 2021). As language learners are exposed to a number of different media, researchers have devoted much attention to understanding the incidental vocabulary learning potentials through learners’ consumption of these media. Specifically, researchers have used lexical profiling to show the incidental vocabulary learning potentials from the listening or viewing of television programs, songs, TED talks, YouTube videos, movies, soap operas, and Netflix series, among others (Webb and Rodgers, 2009a,b; Rodgers and Webb, 2011, 2020; Mohsen, 2016; Nguyen and Boers, 2019; Pavia et al., 2019; Fievez et al., 2021; Ha, 2022b).

This line of research has allowed for a deeper understanding of incidental vocabulary acquisition that occurs in informal settings but at an expense of understanding the potentials for incidental vocabulary acquisition occurring in formal settings. Less attention has been given to the incidental exposure to vocabulary that language learners experience when they receive content instruction through the medium of a second language for which the focus of instruction is on learning content and not language. As second language learners in Macau often spend a large number of hours each day receiving content instruction from teachers that use a targeted second or foreign language (Tam and Reynolds, 2022), it is important for researchers to consider the potentials of this exposure for incidental vocabulary learning.

Higher education institutions in Macau are not unlike others around the world that have language policies that promote the use of English as the language of instruction. However, due to the absence of studies in the Macau higher education context, it then remains unknown whether English instructed higher education in the region provides an adequate opportunity for incidental vocabulary acquisition to occur. Thus, a case study was designed that combined corpus analyses of content course lectures with analyses of a Macau student’s incidental vocabulary acquisition and the student’s perception of the EMI learning experiences after listening to these course lectures. This case study uncovered the potentials of incidental vocabulary acquisition from listening to teacher talk within the Macau higher education context.

### Incidental vocabulary acquisition from teacher talk

Driven by the proposed benefits brought by the internationalization of education, there is an increasing demand for and assumption that content teachers will use English to deliver their courses (Reynolds and Yu, 2018). The implicit assumption is that receiving content instruction in the English language provides a potential opportunity for students’ English vocabularies to develop incidentally through the process of listening to teacher talk (Van Patten, 2003; Horst, 2010). However, only a few studies have tested this assumption empirically within a classroom setting (Smidt and Hegelheimer, 2004; Zhang and Graham, 2020; Brown, 2021).

Examining language related episodes (LRE) occurring inside English medium instruction (EMI) classrooms in two South Korean secondary schools, Hong and Basturkmen (2020) found the majority of the LREs revolved around discipline specific vocabulary. The preoccupation with field specific vocabulary is an indication of its importance to not only understanding content knowledge but also points to the potentials that listening to content lectures have for incidental acquisition of said vocabulary. By contrast, some studies have found listening to teacher talk to be a questionable source for the incidental acquisition of vocabulary (e.g., Horst, 2010; Tang, 2011; Jin and Webb, 2020).

Not all content teachers, especially those from higher education, are willing or able to provide instruction using English. Tang (2011), for example, found the non-native teacher talk of an English teacher in China to be composed of a limited number of words that were also limited in their frequency. These teacher talk phenomena might potentially reduce incidental acquisition outcomes from listening to the lectures. Some teachers may circumvent these issues by increasing the use of first language (L1) translation to encourage the likelihood for incidental acquisition of vocabulary to occur (Jin and Webb, 2020). In addition, an increase in the frequency of exposure to vocabulary during the lectures can further improve this potential (Dang et al., 2022). By contrast, learners’ existing vocabulary knowledge seems to not have as strong of an effect on the likelihood of incidental acquisition. The conflicting results of these studies could be due to their lack of ecological validity as they did not consider incidental learning from listening to lectures for an entire course.

### English as a medium of instruction in Macau

EMI has been a key feature across all levels of Macau’s education. Schools in Macau are authorized to choose a medium of instruction. Macau’s Education and Youth Development Bureau (DSEDJ) has outlined English curricula for EMI and non-EMI schools (namely, schools with Portuguese or Chinese as the medium of instruction) (Macau Education and Youth Affairs Bureau, 2018). The absence of research on the influences of these EMI and non-EMI curricula on students’ English vocabulary development makes it difficult to understand how well students in Macau are prepared by their secondary education for tertiary EMI lectures. These curricula do not specify the expected vocabulary size that students should gain in secondary school and whether this size would be adequate for understanding tertiary level EMI lectures.

Being a multilingual and multicultural region, it is surprising to see a lack of studies looking into how EMI has been implemented in Macau’s higher education. One exception is Yu et al. (2020) that explored undergraduate Mainland Chinese students’ learning strategies while enrolled in EMI courses in Macau. The university’s English for academic purposes courses reported in Yu et al. (2020) did not provide the support needed for the students to excel in the EMI courses. Specifically, the students mentioned the need for more extensive vocabulary instruction. Ma et al. (2022) explored Mainland Chinese postgraduate students’ willingness to communicate in English in the Macau EMI classroom. Ma et al. (2022) found students’ lack of content knowledge led to a decrease in students’ willingness to communicate in the EMI classroom. Rather than fearing that they might lose face as reported in a similar study conducted by Peng (2012), the students were more concerned with their linguistic and content barriers to in-class communication. The findings from these studies indicate that students in Macau are experiencing difficulties in adjusting to the EMI environment.

### Lexical coverage and listening comprehension

Lexical coverage indicates the percentage of words in the input that are known by readers or listeners (e.g., van Zeeland and Schmitt, 2013; Webb, 2021a). For instance, if students know 95 out of the 100 running words in a given text, the lexical coverage figure would be 95%. In recent years, the relationship between lexical coverage and listening comprehension has been the focus of many studies (e.g., Stæhr, 2009; van Zeeland and Schmitt, 2013; Noreillie et al., 2018; Giordano, 2021). Findings from these studies typically suggest contrasting lexical thresholds for adequate listening comprehension. Stæhr (2009) showed that in the case of advanced Danish learners of English who took the Cambridge English C2 Proficiency exam, 90% coverage (i.e., equivalent to a vocabulary size of the most frequent 2,000 word families) only accounted for 55% comprehension of the listening test. To reach adequate comprehension at an advanced level, 98% coverage was required, meaning that students would need to achieve a vocabulary size of the most frequent 5,000 word families. Noreillie et al. (2018) replicated Stæhr (2009) on a different learner population. They found that at an intermediate level, a vocabulary size of the most frequent 1,250 word families (i.e., 91% coverage) would be sufficient for adequate comprehension. Conflicting evidence regarding lexical thresholds for listening comprehension, as discussed in these two studies, is not surprising because comprehension was measured across learner groups of distinct language proficiencies. Nevertheless, as both studies suggest, a larger vocabulary size leads to more successful listening comprehension.

Analyzing lexical coverage in more informal contexts, van Zeeland and Schmitt (2013) found that 90% coverage was sufficient for adequate comprehension of spoken narratives. However, enormous variation in comprehension at this coverage level was observed, meaning that students’ understanding of the texts would be significantly affected by the percentage of the unknown words. At 95% coverage, students were found to achieve a relatively good comprehension. In other words, a vocabulary size of between 2,000 and 3,000 word families would allow them to comfortably understand the spoken texts. Most recently, Giordano (2021) investigated lexical coverage in dialog listening. Participants in the study were at A2-B1 levels based on the Common European Framework of Reference for Languages (CEFR). Students listened to five dialogs at different lexical coverage levels (i.e., 98, 95, 90, 85, and 83%). Similar to the result found in van Zeeland and Schmitt (2013), it was revealed that 90% coverage sufficed for adequate comprehension. Overall, when it comes to lexical coverage necessary for listening comprehension, one would expect a wide range of lexical thresholds from 90% to even 98%, as discussed in previous studies (e.g., Stæhr, 2009; van Zeeland and Schmitt, 2013; Noreillie et al., 2018; Giordano, 2021).

Regarding lexical coverage required for comprehension of academic lectures, to the best of our knowledge, no studies so far have been conducted to examine this relationship. A few studies, however, have examined lexical profiling of academic spoken texts, and the 95 and 98% coverage figures are commonly cited as the starting point of discussion (e.g., Dang and Webb, 2014; Dang, 2022). For example, Dang and Webb (2014) investigated lexical demands in academic English speech across four disciplines. It was shown that a vocabulary size of the most frequent 4,000 word families plus proper nouns and marginal words allows for 96% coverage, while the knowledge of the most frequent 8,000 word families plus proper nouns and marginal words accounts for 98% coverage. Most recently, Dang (2022) analyzed lexical demands in university lectures across EMI courses, non-EMI courses^1^, and open-access non-EMI courses. It was revealed that non-EMI lectures were the most lexically demanding, while EMI lectures were the least. Specifically, to reach 98% coverage, non-EMI lectures require a lexical demand of the most frequent 9,000 word families, while EMI lectures require a lexical demand of the most frequent 7,000 word families. In the case of open-access non-EMI lectures, a vocabulary size of the most frequent 8,000 word families is needed to reach 98% coverage. Following the current practice on lexical profiling (e.g., Dang and Webb, 2014; Dang, 2022), we also use the 95 and 98% cut-off points to examine lexical profiling of the academic lectures collected for the present study. In other words, we want to examine how many words students would need to know to reach 95 and 98% coverage of these academic lectures.

### Assessing incidental vocabulary acquisition

Acquiring a word through incidental exposure is not an all or nothing phenomenon (Laufer, 1998). Instead, acquisition of different aspects of word knowledge occurs from incidental exposure to language input (Nation and Webb, 2011). This necessitates researchers assessing incidental vocabulary acquisition through tests of varied sensitivity (Nation and Webb, 2011); doing so provides a fuller picture of what aspects of vocabulary knowledge can be acquired from the language input under investigation.

The most influential and thorough taxonomy of word knowledge has been proposed by Nation (2013a). Word knowledge can be dichotomously divided into productive and receptive knowledge. When learners possess receptive knowledge of a word, this refers to their ability to understand, comprehend, or recognize a word while possessing productive knowledge refers to their ability to produce the word (Nation, 2013a). The acquisition of the receptive knowledge of words usually precedes the acquisition of productive knowledge (Zhou, 2010). Nation (2013a) also explains that productive and receptive word knowledge can further be categorized into three aspects: form, meaning, and use. Form knowledge refers to knowledge of pronunciation, spelling, and word parts. Meaning knowledge refers to the link between form and meaning, a concept and its referents, and the associations that come to mind when a particular word is presented to a learner. Use refers to grammatical functions, collocations, and constraints (e.g., register). A robust incidental vocabulary acquisition research design incorporates measurements of productive or receptive knowledge for each aspect of word knowledge (i.e., form, meaning, and use). Following the current incidental vocabulary acquisition research practices, we assessed both productive and receptive knowledge in addition to three aspects of word knowledge. Assessing these aspects of vocabulary knowledge provides incidental acquisition results on a spectrum of increasing difficulty, namely receptive knowledge of form, receptive knowledge of meaning, and productive knowledge of meaning.

### Problem statement and research questions

While EMI research conducted in the nearby contexts of Mainland China, Hong Kong, Taiwan, Japan, South Korea, and Vietnam have underscored the needs for student support and teacher professional development (Kung, 2013; Hu et al., 2014; Chen and Kraklow, 2015; Aizawa and Rose, 2019; Tri and Moskovsky, 2019; Hong and Basturkmen, 2020; Macaro and Han, 2020; Sung, 2020; Toh, 2020), it is currently unknown whether Macau higher education is also in need of such support. While previous researchers have acknowledged that it is crucial to evaluate the effectiveness of EMI for content learning and English proficiency development (Peng and Xie, 2021), EMI as input for incidental vocabulary acquisition has largely remained under-researched. The studies that do exist lacked ecological validity as they examined incidental vocabulary learning gains from a severely limited number of lectures that were listened to over a very short period of time. More specifically, there is a need to understand the expected English vocabulary size of incoming undergraduates in the Macau region to determine whether they can gain both content and vocabulary knowledge from EMI lectures. Most of the previous EMI studies used either qualitative research methods to understand in-depth views of stakeholders or quasi-experimental designs to compare the learning outcomes of tertiary students (Bowles and Murphy, 2020; Peng and Xie, 2021); however, quantitative data generated from EMI corpus studies are still largely absent in this growing body of EMI research. The use of corpus analyses allow for the potentials of incidental learning of vocabulary to be examined.

To address the research gaps considered above, the present case study investigated the potentials of incidental vocabulary acquisition from listening to teacher talk from EMI course lectures given at a Macau higher education institution. The following research questions guided the study:

1. How many words do students need to know to comprehend the EMI course lectures?
2. How much vocabulary knowledge can the student incidentally acquire from listening to the EMI course lectures?
3. What is the student’s perception of the EMI course lectures?

## Materials and methods

The method used received ethical review and approval by the Sub-Panel on Social Science and Humanities Research at the University of Macau (SSHRE22-APP014-FED). A case study was conducted because it allows for close scrutiny of a bounded system (Merriam, 1988). In addition, conducting a case study “lay[s] the groundwork for future studies…[on topics for] which little research has been conducted” (Hood, 2009, p. 70). Case study can illuminate the bounded system and the issue under investigation in a way that can inform whether a future large scale study should be conducted.

### Participants

Two participants were recruited for the case study: a university professor and a secondary school student. Convenience sampling was used to recruit the participants. The university professor (the first author) provided recordings of all 27 lectures from an undergraduate *Introduction to English Language Teaching* course he had previously delivered to first-year students at the University of Macau. The secondary school student was referred to the researchers by his English teacher; he was suitable for the study as he was at the end of his secondary school studies and had just received an admissions offer to the Secondary English Education program in which the undergraduate course is taught.

### Data collection

Several types of data were collected for this case study. These included transcripts of lectures given by the university professor, existing vocabulary knowledge of the secondary school student measured by a vocabulary size and levels test, incidentally acquired vocabulary knowledge of the secondary school student measured by researcher-designed vocabulary tests, and interview data about the secondary school student’s incidental vocabulary acquisition experiences from listening to the lectures.

#### Lectures

The lectures were centered on various aspects of language teaching (e.g., native speakerism, intelligibility, instructed second language acquisition, feedback, among others). In 5 out of the 27 lectures, a guest lecturer was invited to interact with the lecturer through a discussion of various topics. The guest lecturer was invited to join several classes because of her knowledge and expertise in the topics under discussion. Regarding her teaching experience and educational background, the guest lecturer has over 10 years of English teaching experience and received her MA and Ph.D. in competitive universities in the United Kingdom. Thus, her English language proficiency would be at C2, the highest level on the Common European Framework of Reference for Languages. At the same time, her speech was perceived as native like by the students enrolled in the course. Accordingly, the inclusion of the guest lecturer would not necessarily affect the analysis of lexical profiling of the current academic lectures. Finally, our corpus data is only made up of academic speech delivered by the lecturers without student interaction. The duration of all the lectures is 15 h and 15 min.

#### Vocabulary knowledge

The student’s vocabulary knowledge was assessed at the start and end of the case study. At the start, the Vocabulary Size Test (Nation and Beglar, 2007) results indicated the student had a vocabulary size of 7,500 English word families and the Updated Vocabulary Levels Test (Webb et al., 2017) showed mastery of the most frequent 3,000 English word families.

The Vocabulary Size Test is a standardized validated test measuring receptive vocabulary size (Beglar, 2010). For the present study, the student was administered the 140-item version of the Vocabulary Size Test with Chinese options and without an *I don’t know* option. While the Vocabulary Size Test is often used in studies that are aimed at measuring breath of L2 English vocabulary knowledge for the purpose of reading (e.g., Zhang, 2013), it has also been shown to be a significant predictor of L2 proficiency (e.g., Leeming, 2014) and operationalized as L2 proficiency (e.g., Reynolds, 2020). While the student’s Vocabulary Size Test results were used as a reference to his English language proficiency, the results of this test have mainly provided information on the student’s receptive knowledge of form-meaning connection of written words.

In contrast, the Updated Vocabulary Levels Test measures receptive vocabulary knowledge for five different frequency bands. It can be used to select words for learning or to ensure that learners are working with learning materials or given language input that is at the appropriate lexical difficulty level. Mastery of vocabulary levels was operationalized as answering 29 out of 30 of the items correctly on the 1,000, 2,000, and 3,000 levels and 24 out of 30 of the items correctly on the 4,000 and 5,000 levels of the Updated Vocabulary Levels Test. In other words, the student answered at least 29 of the 30 items on level 1 (30/30), level 2 (30/30), and level 3 (29/30) but was unable to answer 24 out of 30 items on level 4 (23/30) and level 5 (21/30) of the Updated Vocabulary Levels Test. The 29/30 cutoff for the most frequent 3,000 word families of English was suggested by the developers of the Updated Vocabulary Levels test “as these words account for such a large percentage of English [and] they provide the foundation for further lexical and language development” (Webb et al., 2017). It was important to pay close attention to these words because mastery of the most frequent 3,000 word families of English may account for as much as 95% of spoken discourse. Some researchers have claimed mastery of these 3,000 words sufficient for the comprehension of spoken input (van Zeeland and Schmitt, 2013). As the items that appear on the Updated Vocabulary Levels Test and the corpus analyses reported in the present study (see Section “Corpus analyses”) draw on the same lexical sources, using the student’s results on the Updated Vocabulary Levels Test together with the corpus analyses provides an estimate of the words the student was likely to have known while listening to the lectures.

Measuring incidental gains in different aspects of word knowledge required the construction of three assessments at different levels of sensitivity (Nation and Webb, 2011) (see Supplementary Material). The pre-test and post-test versions of the assessments contained the same items but were presented in a different order to reduce the likelihood of practice effects. Distractors for the receptive form assessment were constructed with the assistance of Wuggy (Keuleers and Brysbaert, 2010), and semantically-related distractors for the receptive meaning assessment were constructed in English before being translated into Chinese, the first language of the student; Chinese distracters were used to ensure that incidental acquisition was being assessed and not comprehension of the items. An *I don’t know* option was included in each meaning recognition assessment item to discourage guessing (Nation and Webb, 2011). The addition of an *I don’t know* option has been shown to reduce the likelihood of guessing (Zhang, 2013). Items on the three word knowledge assessments were marked as either correct or incorrect. The productive meaning assessment was marked independently by the first and second author showing a 100% intermarker agreement. The multiple choice items for the receptive form and receptive meaning assessments were marked using a key.

#### Target words

Prior to target word selection, we set a number of criteria. Firstly, given the student’s vocabulary size and knowledge, we selected words that occurred within the 6,000 frequency level and beyond. This reduced the chance that a target word would be known to the student at the start of the study. Secondly, the target words must be content words that appeared at least 5 times in the corpus. Previous research has shown five encounters in auditory input to allow inferring of partial word knowledge (Webb, 2007; Webb and Rodgers, 2009b). Thirdly, selected words must have a relatively similar length in terms of letters and syllables. This will help reduce variation in the difficulty level of the words, thereby improving the reliability of the results. In the end, 10 target words were selected (see Table 1 for a complete profile of the target words). Finally, to ensure that the target words were unknown to the student, we sought professional consultation from his high school teacher, who later confirmed that the student was less likely to know these target words.

**TABLE 1:** Profile of the target words.

Target words	Part of speech	Letters	Syllables	Frequency of occurrence	Frequency level
Cognate	Noun	7	2	5	13,000
Recast	Verb	6	2	11	10,000
Lexical	Adjective	7	3	35	8,000
Fallacy	Noun	7	3	7	8,000
Syntax	Noun	6	2	8	8,000
Scaffold	Verb	8	2	37	7,000
Normative	Adjective	9	3	6	7,000
Salient	Adjective	7	3	9	6,000
Increment	Noun	9	3	8	6,000
Holistic	Adjective	8	3	5	6,000

#### Interview

The first author conducted a 60 min semi-structured interview with the student in English (at the student’s request) at the end of the study after he had listened to all of the EMI course lectures (see Supplementary Material). English was chosen because of the student’s high motivation to engage in English speaking activities. The first author had established a close relationship with the student through bi-weekly contact when providing the student with the recorded lectures. This established comradery made it possible for the student to freely and honestly express his views during the interview.

### Procedures

**FIGURE 1:**
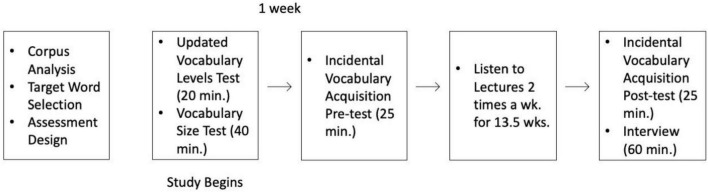
Research procedures.

The procedures are schematized in Figure 1. Prior to the start of the study, the researchers completed the corpus analyses, selected the target words, and designed the vocabulary assessments. Then on the first day of the study, the student was administered the updated vocabulary levels test followed by the vocabulary size test. Then 1 week later, the student was administered the incidental vocabulary acquisition pre-test and then instructed on how to listen to the lectures. The student listened to each lecture a single time (two lectures a week–1 on Friday and one on Sunday) for the next 13.5 weeks. We provided the student access to the lectures one at a time with Google Drive by providing him viewer privileges for a 24 h time period. He was asked to set aside the same time on Fridays and Mondays to listen to each lecture from start to finish without rewinding or fast forwarding. This aimed to simulate a regular course schedule. The student was asked to use the Google Drive web interface to listen to the lectures without rewinding or fast forwarding. He used a Windows 11 compatible desktop computer with a 22″ monitor and over-ear headphones to listen to the lectures. At the end of the study an interview was scheduled but on the day of the interview and unbeknownst to the student, the post-test was administered prior to conducting the interview. The post-test was not made known to the student beforehand as this could potentially encourage intentional learning of vocabulary while he listened to the lectures (Nation and Webb, 2011).

### Corpus analyses

To begin the data cleaning process, all the lectures were transcribed using the *Dictate* function in Microsoft Word. Non-verbal actions (e.g., *cough* or *laugh*) were removed since they did not contribute to the lexical demand. Following this was thorough examination of the transcribed lectures to correct misspelling errors made by the transcription software. In the current study, Nation’s (2017) British National Corpus (BNC)/Corpus of Contemporary American English (COCA) 25 1,000-word family lists were utilized, with the supplementary four lists of proper nouns, marginal words, compounds, and acronyms. Words that are not found in the wordlists were classified as off-list words. To comply with the spellings used in the wordlists, contractions (e.g., *shouldn’t* or *mustn’t*) were written in full form, while hyphenated words (e.g., *task-based* or *so-called*) were de-hyphenated. In the case of spelling (e.g., L-E-M-M-A), hyphens were removed, but each letter was written separately (e.g., L E M M A) because it reflected how students perceived the word being spelled in the lectures. Phonetic transcriptions such as/s/or/t/were also taken out since students are likely to recognize them due to their high frequency (Dang and Webb, 2014). Furthermore, in our corpus, only five phonetic transcriptions were found, and thus, the removal of phonetic transcriptions would not significantly affect the analysis. After the data cleaning process, the present corpus consists of 128,498 tokens and 3,952 types. The average speech rate of all the lectures is 140 tokens per minute, which was deemed appropriate for academic lectures, according to Tauroza and Allison (1990).

Prior to data analysis, all lectures were converted into text files. Subsequently, AntWordProfiler (Anthony, 2022) was employed to examine lexical demand of the entire corpus. In the first round of the data analysis, the program identified new proper nouns (e.g., *Moodle* or *WeChat*) and marginal words (e.g., *ahchoo*) that were not listed in the original wordlists. To ensure a more precise calculation of lexical demand of the lectures, these words were added to the original wordlists. In the current study, proper nouns and marginal words were counted in the analysis of lexical coverage on the assumption that they pose a slight learning burden on students (Nation, 2006; Dang, 2020). In other words, it was assumed that students would likely recognize them in lectures. In the case of compounds and acronyms, our data analysis showed that the present corpus contains several uncommon compounds (e.g., *milestone* or *sociocultural*) and an array of professional acronyms (e.g., *CLT* or *CLIL*). Hence, they were excluded from the analysis of lexical coverage on the grounds that students might be unaware of their meanings. At the same time, the proportions of compounds and acronyms were found negligible, accounting for 0.4 and 0.14%, respectively, and thus, their exclusion was thought not to affect the result. Finally, lexical profiling of each lecture was also reported to provide a more comprehensive picture of lexical demand across different lectures.

The current corpus is made up of academic lectures. Thus, we adopted word family as the counting unit in our study since it is considered as a suitable and useful approach to deal with receptive knowledge (i.e., listening comprehension) (Nation and Webb, 2011; Vilkaitë-Lozdienë and Schmitt, 2019). Following this, we counted the frequency of the target words by word family. In other words, occurrences of all variants of a word would be counted (Reynolds and Wible, 2014). For example, if *colony* occurred 4 times, *colonial* 1 time; *postcolonial* 2 times; *colonizing* 3 times, the cumulative frequency of occurrence of the word *colony* would be 10 times.

### Content analysis

Content analysis (Drisko and Maschi, 2015) was used to analyze the student’s responses to the semi-structured interview questions (see Supplementary Material). Collaboration between co-authors increased the reliability and quality of the analysis (Robson, 2011). The audio recording of the interview was transcribed by a research assistant using the *Dictate* function in Microsoft Word before the research assistant corrected transcription errors. This transcript was then further proofread and corrected by the first and second author. Given the exploratory nature of this qualitative data, inductive coding of the interview transcript was completed in four stages. In stage one, the first and second author became familiar with the data by reading and rereading the transcript. Then, in stage two, the second author loaded the transcript into NVivo Software ver. 1.3.1 to generate initial data-driven codes. In stage three, the first author verified the codes and suggested changes to the second author before the codes were finalized and approved by the first and second author. In the last stage, the codes were combined to form themes that represented the data.

## Results

Our data analysis showed that the majority of tokens (i.e., the occurrence of a word), types (i.e., the unique occurrence of a word regardless of its frequency), and word families (i.e., all variants of a word) were found in the list of the most frequent 1,000 word families. Specifically, 109,952 out of 128,498 tokens, 1,513 out of 3,952 types, and 710 out of 2,190 word families come from the 1,000 frequency level list (see Table 2). The finding would come as no surprise given the spoken nature of the current corpus.

**TABLE 2:** Tokens, types, and word families at each word level for the entire present corpus.

Word list	Tokens		Types		Word families	
	Raw	%	Raw	%	Raw	%
1,000	109,952	85.57	1,513	38.28	710	32.42
2,000	8,541	6.65	885	22.39	414	18.9
3,000	5,084	3.96	776	19.64	422	19.27
4,000	680	0.53	186	4.71	139	6.35
5,000	757	0.59	99	2.51	78	3.56
6,000	367	0.29	59	1.49	45	2.05
7,000	259	0.2	49	1.24	37	1.69
8,000	96	0.07	32	0.81	29	1.32
9,000	18	0.01	10	0.25	10	0.46
10,000	41	0.03	19	0.48	14	0.64
11,000	47	0.04	15	0.38	14	0.64
12,000	15	0.01	6	0.15	5	0.23
13,000	21	0.02	12	0.3	11	0.5
14,000	21	0.02	7	0.18	5	0.23
15,000	51	0.04	5	0.13	5	0.23
16,000	8	0.01	4	0.1	4	0.18
17,000	37	0.03	5	0.13	5	0.23
18,000	2	0	1	0.03	1	0.05
19,000	0	0	0	0	0	0
20,000	4	0	3	0.08	3	0.14
21,000	0	0	0	0	0	0
22,000	0	0	0	0	0	0
23,000	2	0	1	0.03	1	0.05
24,000	0	0	0	0	0	0
25,000	1	0	1	0.03	1	0.05
Proper nouns	1,589	1.24	145	3.67	129	5.89
Marginal words	90	0.07	10	0.25	8	0.37
Compounds	512	0.4	40	1.01	33	1.51
Acronyms	179	0.14	16	0.4	14	0.64
Off-list words	124	0.1	53	1.34	53	2.42
Total	128,498		3,952		2,190	

In answering RQ1, it was found that a vocabulary size of the most frequent 3,000 word families plus proper nouns and marginals words allows students to understand 97.49% of the entire corpus, while they will need to know the most common 4,000 word families plus proper nouns and marginal words to comprehend 98.02% (see Table 3). Further analysis, however, showed variations in lexical demand across individual lectures (see Supplementary Material). Specifically, to reach 98% coverage, nine lectures required a lexical demand of 3,000 word families, five lectures required a lexical demand of 4,000 word families, ten lectures required a lexical demand of 5,000 word families, and one lecture required a lexical demand of 6,000 word families. There were two lectures (lecture 21 and lecture 22 in our corpus) for which a vocabulary size of the most frequent 25,000 word families would only allow for 96–97% coverage. The reason for this is that our analysis excluded compounds which happened to account for a high percentage in these two lectures (3.67% in lecture 21 and 2.15% in lecture 22). Another intriguing finding was that the proportion of proper nouns were particularly large in lecture 9 and lecture 15, accounting for 5.52 and 4.18% in turn, as opposed to their low percentages in the other lectures.

**TABLE 3:** Lexical coverage for the entire present corpus (%) with proper nouns and marginal words.

Word list	Coverage at each 1,000-word level without proper nouns and marginal words	Cumulative coverage with proper nouns and marginal words
1,000	85.57	86.88
2,000	6.65	93.53
3,000	3.96	97.49^a^
4,000	0.53	98.02^b^
5,000	0.59	98.61
6,000	0.29	98.9
7,000	0.2	99.1
8,000	0.07	99.17
9,000	0.01	99.18
10,000	0.03	99.21
11,000	0.04	99.25
12,000	0.01	99.26
13,000	0.02	99.28
14,000	0.02	99.3
15,000	0.04	99.34
16,000	0.01	99.35
17,000	0.03	99.38
18,000	0	99.38
19,000	0	99.38
20,000	0	99.38
21,000	0	99.38
22,000	0	99.38
23,000	0	99.38
24,000	0	99.38
25,000	0	99.38
Proper nouns	1.24	
Marginal words	0.07	
Off-list words	0.1	
Tokens	128,498	

^a^Reaching over 95% coverage, ^b^reaching over 98% coverage.

In answering RQ2, the pre-test results showed the student had receptive form knowledge of four targeted words, productive meaning knowledge of zero targeted words, and receptive meaning knowledge of one targeted word. Incidental vocabulary acquisition gains were calculated by subtracting the pretest scores from the posttest scores. The student showed the following *gains* in lexical knowledge: receptive form for two words, productive meaning for one word, and receptive meaning for four words.

Seven themes were generated to answer RQ3. The themes have been placed into three groupings: the student’s perception about listening to EMI lectures (see Table 4); the student’s perceptions about listening to EMI lectures for vocabulary gains (see Table 5); and the student’s perceived needs and suggestions for teachers (see Table 6).

**TABLE 4:** The student’s perceptions about listening to EMI lectures.

Theme	Codes
Benefits of listening to the EMI lectures	Content learning
	Vocabulary gains
Difficulties when listening to the EMI lectures	Difficulty with engagement while listening
	Difficulty in understanding due to a lack of language proficiency
	Difficulty in understanding due to a lack of content knowledge
	Difficulty in understanding due to native speaker lecturer’s language use
	Vocabulary and learning strategy used
	Lack of secondary school English training in understanding EMI lectures

**TABLE 5:** The student’s perceptions about listening to EMI lectures for vocabulary gains.

Theme	Codes
Possibility for vocabulary gains	Source for vocabulary gains – vocabulary used related to the EMI lecture topics
	Teacher talk strategy aided vocabulary gains – explanations
Reasons for reduced possibility for vocabulary gains	Easier vocabulary used
	Not essential vocabulary for understanding content
The perceived influence of vocabulary gains	Teacher’s explanations
	For future study in an EMI environment
	For future teaching career

**TABLE 6:** The student’s perceived needs and suggestions for teachers.

Theme	Codes
Suggestions for university EMI lecturers	Consider students’ varied language proficiency levels and needs
	Students need to use L1 and translanguaging in EMI courses
	Potential impact of using L1 and translanguaging in EMI courses
	Strategies to aid vocabulary learning – additional readings and videos
	Teacher talk strategies can enhance EMI learning experience – interaction in the classroom
Suggestions for secondary school English teachers	Motivate students to learn English
	Raise students’ awareness about university EMI lectures
	Use readings and videos as teaching materials to prepare students for university EMI lectures

The student claimed two benefits of listening to the EMI lectures. He found the lectures helpful for learning content knowledge (e.g., “th[e] information …. in these videos are very useful …. [to] my future …. lessons later in the university… Very useful for those who are going to study English education.) and for gaining vocabulary knowledge (e.g., “I learn[ed] some English vocabulary terms … expressions…”). By contrast, he mentioned six difficulties in listening to the EMI lectures. As his English lessons in high school differed from the EMI lectures, he found it difficult to stay engaged while listening to the lectures (e.g., “one of the difficult[ies] … is to … focus on the video for … an hour …). He found his language proficiency and content knowledge not sufficient (e.g., “I do not understand the content of [all] those videos … I [am] aware … that … my English is not … good enough to … understand everything in … those videos…”), thereby making listening to the lectures difficult for him. While he claimed to be a highly motivated student, the potentials of incidental vocabulary acquisition could have been affected by the reduced attention as well as the lack of content and vocabulary knowledge.

The student also held an opinion about the language used by the English native-speaking lecturer. He claimed that the way that his non-native English-speaking teachers gave EMI lectures in high school was different from the way the native-speaking English teacher gave EMI lectures in the recordings. His high school teachers “tend[ed] to … say everything in an easier way … [and] those people [in the lectures] sp[o]k[e] in a more difficult way.” He presented a rather contradictory view of the EMI lectures as containing language that was spoken “in a more difficult way” but could provide him opportunities to “understand more” because the lecturer was willing to explain the new concepts and vocabulary. While he perceived these newly encountered vocabulary as “professional” they were still considered “difficult” as he had never heard them before. He would “guess the meaning from what the person [was] saying,” an example of an incidental vocabulary acquisition strategy. He felt this strategy was not always effective as applying it only resulted in his understanding of around 80% of what was spoken in the lectures. He also shared that his secondary school English teachers did not prepare him to understand university EMI lectures. He claimed “the feeling [of listening to the recorded lectures was] not as familiar as taking lessons in [secondary] school.”

The student held perceptions about listening to EMI lectures for vocabulary gains. He perceived the lectures as sometimes enhancing and at other times reducing the opportunities for incidental learning. He shared his opinion on what variables might have influenced the uptake of new vocabulary knowledge. The themes and their corresponding codes are provided in Table 5.

There were two reasons given for why he learned vocabulary from listening to the lectures. First, he mentioned that the words most important to the topic of the lectures were focused on more by the lecturer so this added attention given to them by the lecturer left a lasting impression on him. For example, he shared “another word called Lingua Franca … I remember the meaning … treating a language as the first language … there is Hong Kong English or Singapore English … I have gained a lot more vocabular[y] … mostly concept[s].” He was able to recall several words and concepts that he learned about from listening to the lectures. He also shared that he was able to pick up the terms because “the speaker … explain[ed] the terms or words very clearly … [and] it’s very important to make sure that the students will understand the content.”

The student also shared that he sometimes did not learn new words from listening to the lectures because the words spoken were ones he already knew or he felt were not important to understand the content of the lectures. He would just ignore them. He explained that “there were words that were difficult, but I …. d[id] not spend a lot of time or effort to … get the[ir] meaning because I th[ought they] were not … vital to … understand … the topic that [was] being introduced.”

When further queried on what factors might have affected the incidental learning of vocabulary encountered through listening to the lectures, he said that incidental learning could have been affected by the teacher’s explanations of the unknown words, whether he felt the unknown words were useful for his future university studies, and whether the vocabulary would be useful to his future career goal of becoming a secondary school English teacher. He explained that if the lecturer did not “explain very … comprehensively, [he would] not have a very good impression about the word.” When he came across words, especially concepts related to English education, his attention to the lectures would be heightened as he felt listening to these particular parts was “getting [him]self more prepared for the things [he] will learn in the university … [and he] might need to use those concepts or ideas that are introduced in those videos … when [he] becomes … [an] English teacher.”

The student also provided suggestions to both his future university lecturers and his previous secondary school English teachers. He provided five pieces of advice for university lecturers and three pieces of advice to secondary school English teachers (see Table 6).

The student said that university EMI lecturers should be aware that the language proficiency “level of students varies.” This means some students may need more scaffolding than others to understand the content. This thought caused the student to consider whether he could have learned more content knowledge from the lectures if the lectures had been delivered in Chinese, his first language. He shared that “it might be more effective … if … Chinese [was used] to express the meaning, because [he] might have a stronger impression and a better understanding of those vocabulary … [This] might make it more … efficient and more effective…”. The student explained an internal struggle he experienced when thinking about whether English or Chinese should be used in higher education. He concluded his answer by saying that lectures “in English will [provide] more improvement in … English … [and lectures] in Chinese … might not [result in] good English.”

In order to provide students the opportunity to improve their English through listening to English lectures while also taking care of content knowledge needs, he suggested university lecturers could provide additional reading materials and videos to students to review before listening to lectures. He assumed that when he took courses in the future that the lectures would be accompanied by “passage[s] or book[s]” and that he could “search for the meaning of the words that [he] did not understand to … [understand] the content more thoroughly.” The discussion of what might occur in a future EMI classroom led to his sharing that he might have learned more vocabulary if there was interaction among the lecturer and students. For this study, he only listened to the lectures but in a formal classroom he would not only listen to lectures but also have the opportunity to interact with classmates and offer questions to the lecturer for clarification. He felt that listening to lectures for this study:

… must be more difficult than having lessons in the classroom because … I am just receiving things … [and] not able to ask question[s]. I [can’t ask] the speaker to elaborate [on] something or to further explain something … that’s one of the shortcoming[s] of [listening to] these lectures.

He felt that in the regular classroom, a lecturer would increase the opportunities for incidental acquisition of vocabulary simply by allowing students to ask questions.

The student also offered three suggestions to secondary school English teachers to help prepare students for the experience of listening to EMI lectures in university. He felt that by sharing with the students what they are going to experience in university, the secondary school English teachers could motivate students to put more effort into their English studies. They could try to provide them with examples of lectures such as the ones he viewed so that students would know what to expect and would not have the same assumptions as he had before this experience. He felt that this could help “the students’ mindsets prepare [for] how it will be like in … the university.” The teachers could also try to use such materials as the content in their English classes to gradually get the students familiar with content learning in English.

## Discussion

Our findings showed that a vocabulary size of the most frequent 3,000 word families plus proper nouns and marginal words enables students to understand 97.49% of all the present EMI lectures combined. If they know the most frequent 4,000 word families plus proper nouns and marginal words, they can understand 98.02%. However, Dang (2022) found that to reach 98% coverage of EMI lectures, students would need to achieve knowledge of the most frequent 7,000 word families. Clearly, there was a huge difference between the lexical demands for our corpus and the corpus presented in Dang (2022). Such a difference can be attributed to many factors. First, the size of our corpus is significantly smaller than the size of the EMI corpus in Dang (2022), 128,498 and 253,906 words, respectively. Clearly, the corpus of a bigger size would contain significantly more new words, resulting in a greater lexical demand, especially at 98% coverage. Secondly, our corpus data is derived from one single discipline, which is *English Language Teaching*; however, the EMI corpus in Dang (2022) consists of various disciplines. This could account for variations in lexical demand across EMI lectures of different disciplines. Another potential factor is the delivery of the lectures. It could be that lexical usage in our corpus was intentionally modified by the lecturers to facilitate students’ understanding of the new concepts and ideas. This helps explain why lexical demand at 98% coverage was found to be lower in our study compared to what was found in Dang (2022). Undoubtedly, this claim is tentative, but it provides an important pedagogical implication in that the analysis of lexical demand of academic lectures should be contextualized within a specific classroom. Overall, we want to emphasize that only through the examination of lexical demand of the lectures collected in a specific course can we provide students with a more precise and realistic estimation of how many words they need to know to reasonably understand the lectures. This is because lexical coverage of academic lectures can be affected by various factors, such as the duration of the lectures, the disciplines, or even the form of delivery.

Variations in lexical demand across individual lectures were also observed in our study. Specifically, ten lectures required students to possess a vocabulary size of the most common 5,000 word families to comprehend 98% of the texts, and one lecture required a lexical demand of 6,000 word families to achieve this same level of understanding. Pedagogically, we suggest that in the case of more linguistically complex lectures, teachers should introduce students to novel words in advance (e.g., having them do a vocabulary quiz in class or asking them to read the textbook materials before class). This would maximize students’ understanding of the subject content.

Our study excluded compounds from the analysis of the lexical coverage of the current corpus on the grounds that several compounds were found to be advanced lexical items and could create learning difficulties for students. However, analysis of individual lectures showed that two out of the twenty-seven lectures consisted of a high proportion of compounds. Pedagogically, our finding suggests that in the case of infrequent compounds, teachers could introduce them to students beforehand to improve students’ understanding of the lesson content. Methodically, our finding raises an important question in research on lexical coverage, that is, how to best deal with compounds (e.g., whether to include or exclude them from the lexical coverage analysis). Cobb (2019) proposed an interesting approach that compounds should be first broken down (e.g., *shutdown* would be broken into *shut* and *down*), and then their pieces will be put back to the wordlists for analysis. Breaking compounds down, however, would eventually increase the number of words in the corpus, thus affecting the calculation of the speech rate. Clearly, more research on how to effectively deal with compounds in the calculation of lexical coverage is warranted.

In the case of proper nouns, they are usually included in the analysis of lexical coverage based on the assumption that proper nouns pose a slight learning burden on students (e.g., Nation, 2006; Webb and Rodgers, 2009b; Dang, 2020). In our study, the proportion of proper nouns accounted for 1.24% of the entire corpus. However, this percentage is immensely large in certain lectures. Specifically, the proportion of proper nouns is even higher than the coverage of the 3,000-word level list in lectures 9 and 15. Kobeleva (2012) found that unfamiliar proper names can significantly affect students’ listening comprehension. Pedagogically, we recommend that if a particular lecture contains a large number of unfamiliar proper nouns, teachers should pre-teach them to aid students’ understanding of the lectures. Likewise, it might be unreasonable to expect students without mastery of the most frequent 3,000 words of English to comprehend lectures from this course. Methodologically, our study raises an important question as to whether unfamiliar proper nouns should be included in the calculation of lexical coverage given that they can pose difficulties to students when they listen to academic lectures.

The student’s vocabulary knowledge should have provided him almost 98% lexical coverage of the lectures. This seems ideal for encouraging incidental vocabulary acquisition; however, after listening to 15 h and 15 min of lectures, the student gained receptive knowledge of form for two words, productive knowledge of meaning for one word, and receptive knowledge of meaning for four words. These results may seem very modest but it must be noted that the student is likely to have acquired knowledge of words that were not targeted in the present study. Certainly, various factors came into play and affected the incidental vocabulary learning from listening to academic EMI lectures. While it is difficult to pinpoint these variables, frequency of exposure and salience are likely to have contributed to the incidental acquisition of word knowledge.

Frequency of exposure has been shown to be a robust predictor of incidental vocabulary acquisition (e.g., Rott, 1999). The frequency effect seems to have resulted in the student acquiring the productive knowledge of meaning for one targeted word (i.e., scaffold). This word was spoken 37 times by the lecturer, the most frequently spoken among the 10 targeted words. It is not surprising to have found the student to have only acquired the productive knowledge of meaning for only one of the ten targeted words as productive knowledge is more difficult to acquire than receptive knowledge (Nation, 2013a). This is because word details must be attended to when a person speaks or writes during language production (Webb and Nation, 2017). As there was no opportunity for the student to use these words productively during the study, having acquired little productive knowledge of vocabulary comes as no surprise. The implications for content teachers is that without a flood of exposure to unknown words, they should not assume that students will have the wherewithal to incidentally acquire the knowledge of unknown words to the point that they can be used productively. As it might not be feasible for a teacher to increase the frequency of exposure to the words through repetition in their lectures, an alternative would be to ensure important words are used when completing coursework. Having students complete tasks that require language output (i.e., speaking and writing) will result in more incidental vocabulary learning than completion of receptive tasks (i.e., listening and speaking) (Webb, 2005).

The effect that frequency has on the learning of vocabulary cannot be considered in isolation. The target word *lexical* was spoken 35 times by the lecturer; however, the assessments did not show the student to have acquired productive knowledge of this word. The word *scaffold* was spoken in six lectures and the word *lexical* was spoken in four lectures. Previous research has shown that the knowledge of words that are more evenly dispersed across language input as being more likely to be incidentally acquired (Reynolds and Ding, 2022). Furthermore, looking at the occurrences of the word *scaffold* show it was spoken by the lecturer most often during lecture 1 (*n* = 11) and lecture 2 (*n* = 19) before being spoken again during lectures 19, 20, 21, and 22. Research has also shown knowledge of new words are better retained if they are first presented in a massed learning condition before being followed up with a spaced learning condition (Uchihara et al., 2019). This is because the initial massed learning allows language learners to more easily make a form-meaning connection, the initial step necessary for vocabulary learning (Nation, 2013a). Thus, the accumulated effects of frequency, range, and massed learning followed by spaced learning resulted in the productive knowledge of meaning for the word *scaffold* being incidentally acquired by the student. In contrast, the lecturer spoke the word *lexical* for the first time in lecture 17 (*n* = 2) and not in a massed learning condition, potentially reducing the chance for the student to make the form-meaning connection. By lectures 25, 26, and 27 the word *lexical* did occur in a massed learning condition but the study had just ended, eliminating the possibility of a follow up spaced learning condition.

Language learners often perform better at receptive knowledge assessments as they only need to recognize a word passively in language input (Webb and Nation, 2017). This was also found in the incidental vocabulary acquisition post-test results of the student. He showed receptive knowledge of form gains of two words and receptive knowledge of meaning gains of four words. While the difficulty in productive knowledge assessments can partially explain the difference in the incidental acquisition outcomes between the productive and receptive assessments, it is likely salience was more influential than frequency. This is because the frequency of exposure through the lectures for the acquired words varied considerably. Thus, it is likely that for one reason or another the incidentally acquired words were found to be more salient to the student. Studies involving the incidental acquisition of reading have found as few as three exposures were needed for incidental acquisition to occur if the task was found important to the students (Reynolds, 2015a). Salience can be encouraged in a number of ways but most likely it was due to the student’s perception of the importance of the words or the lecturer’s emphasis or explanation of the words. Another likely possibility was the context in which the repetitions of the words were very informative, providing an abundance of explication that allowed for the learner to pick up the meaning incidentally (Webb, 2008; Soyoof et al., 2022).

Out of the ten targeted words, eight were explicitly explained by the lecturers and two were not. The words *scaffold* and *lexical* were spoken by the lecturers 37 and 35 times, respectively. The assessment results showed the productive knowledge of meaning of *scaffold* was incidentally acquired by the student, but *lexical* was not. The reason may be that *scaffold* was presented as an educational concept that was first defined and then exemplified. On the contrary, *lexical* was spoken as a part of 11 two-word phrases or chunks rather than a single word, such as *lexical demand, lexical coverage*, and *lexical chunk*. Seven out of 11 of these two-word phrases were not explained by the lecturers. Accordingly, the increasing exposure to the word *lexical* may not have increased the student’s chance of incidentally acquiring the word as explanations or definitions were not provided for many of these two-word phrases. Hence, even though the exposure through listening to the lectures was relatively frequent, they may not have been considered as recurrences of the same word by the student (Reynolds, 2015b).

The EMI lecturers used the following teacher talk strategies to aid listeners’ understanding of the target words: giving examples of use, providing definitions, describing scenarios, providing synonyms, paraphrasing meaning, and highlighting the purpose of their use. Regarding the quality of explanations offered by the lecturers, most of the words were explained with clear definitions and examples, while two words were only spoken in the lectures without explicit definitions or examples. Although definitions and examples were used as supportive strategies for explanations, in some cases, we tend to feel that overly explaining the words might make simple concepts complicated. In particular, the lecturers explained *fallacy* and *recast* by providing demonstrations that were lengthy and potentially construed as difficult to follow, especially for a first-year first-semester English education major. This might have reduced the frequency effect and thereby reducing the likelihood of the student to have incidentally acquired these words. The implication for content teachers is that quality of contexts and introduction of new vocabulary is important for incidental learning. Teachers are advised to provide high quality definitions, illustrations, and examples when introducing new concepts in their lectures.

Although the student reported several difficulties in listening to the EMI lectures, he also reported the experience as satisfying. He felt this experience helped prepare him for future content learning in the university. This result supports Reynolds and Ding (2022) that reported participants were able to incidentally acquire vocabulary from completion of a task even when the task was found to be difficult. The student felt the difficulties he faced were due to his English proficiency and unfamiliarity with the course content. A similar situation was reported by Ma et al. (2022, p. 96) that found “linguistic and content barriers” prevented Mainland Chinese graduate students studying in Macau from getting the most from their EMI lectures. These are examples of barriers that Peng and Xie (2021) claimed that should be addressed when implementing an EMI policy in schools. It is necessary for schools in Macau to consider what types of support they should provide to students in the period of adjustment as they transition from a non-EMI to an EMI environment. University lecturers should also be aware that many of the students may have inadequate vocabulary knowledge to understand all of their lectures and should be prepared to provide proper scaffolding to ensure effective instruction.

The finding that the student was able to incidentally acquire vocabulary from listening to academic English lectures has given support to previous findings about the use of academic lectures as language input for vocabulary learning (Dang et al., 2022). The student claimed his interest in the lecture topics and their relevance to his future studies and work is what really honed his attention on particular concepts and vocabulary, thereby inducing a state of incidental learning. He also mentioned that the difficulty of understanding particular concepts was reduced by the lecturer’s explanations, thereby highlighting the importance of providing training to lecturers in how to facilitate their students’ incidental vocabulary acquisition using relevant teacher talk strategies (Horst, 2010).

The student’s interview responses highlighted the need for a bridge between secondary and tertiary education. His experience in listening to the EMI lectures underscored the lack of preparation he felt he received from his secondary school English teachers. If given similar opportunities to listen to EMI lectures in secondary school, he thought that would have been a strong motivator for him and his peers to have paid more attention in English class. Likewise, he felt his secondary school English teachers could have used some teaching materials that mimicked those that would be provided in tertiary education instead of only providing some simple reading materials or language exercises. He assumed when formal classroom lecturing occurred in the university that he would be provided opportunities to interact with his teachers and peers. He had the assumption that these interactions would facilitate the incidental acquisition of vocabulary encountered in class. His assumption has actually been supported in previous research by Hong and Basturkmen (2020) that found most LREs initiated by the students in their study were about field specific vocabulary. Thus, providing such opportunities to students in the EMI courses is highly recommended.

While English for academic purposes courses surely can provide academic support to students, they may not be enough to properly prepare students for EMI lectures. Candid explanations regarding the benefits and potential drawbacks of EMI should be discussed with not only the students but also the lecturers. Teacher development courses that expound Macau’s educational context, the status of the English language, the overview of students’ linguistic knowledge, capacity for EMI learning, and needs, among others, can allow lecturers to better facilitate their students’ content learning. Such training can help maintain quality EMI programs in Macau (Xie, 2021).

Without such open discussions, the student held an unfounded belief that non-native speakers of English would provide EMI lectures that were easier to understand than lectures provided by native speakers of English. He felt non-native speakers of English are better equipped to understand the needs of non-native speaking students. For example, he said they could easily provide on-the-fly first language translations for unknown words. While limited use of the first language in EMI lectures could help students understand the content being delivered (Yu et al., 2020) and quickly provide translations for thorny concepts or vocabulary (Jin and Webb, 2020), this is not an option for lectures that do not share a common language with the students. Thus, he assumed that native speakers, regardless of their training, educational backgrounds, or experiences, would deliver lectures that were more difficult to understand. The student also struggled with his belief that attending lectures delivered in English would help improve his English language but might prevent him from fully understanding the content.

While these are all valid concerns, some of them could have been alleviated through new student orientation. Therefore, not only the lecturers should be provided with professional training but also students should be given proper transitional support and orientation that ensures they fully understand this new educational context. These findings coincide with the existing studies that have found varied forms of student support along with teacher professional development can lead to successful EMI educational outcomes (Chen and Kraklow, 2015; Aizawa and Rose, 2019; Hong and Basturkmen, 2020; inter alia).

## Conclusion

The findings of this case study have implications for Macau higher education. First, the results found a vocabulary size of the most frequent 4,000 word families plus proper nouns and marginals words allows students to understand 98% of the entire corpus composed of lectures from the *Introduction to English Language Teaching* course. Similar techniques could be applied to other content course subjects besides English language teaching to determine whether mastery of the most frequent 4,000 word family is also adequate for comprehension of EMI course lectures given in Macau’s higher education institutions. While modest, the participant was able to incidentally acquire vocabulary knowledge from listening to the lectures. Still, institutions of higher education in Macau cannot assume students without adequate vocabulary knowledge will be able to pick up most of this knowledge incidentally by listening to EMI lectures. As this study’s corpus analyses show, some of the lectures presented a lexical burden that might have reduced the likelihood of incidental vocabulary learning. Instead, teacher talk may need to be adjusted, and other pedagogical strategies should be applied to increase the likelihood of incidental learning. To enhance students’ content learning and incidental vocabulary acquisition through the listening to EMI lectures, EMI lecturers are advised to use varied strategies while explaining the target concepts and words to students.

The student’s responses to the interview questions resulted in seven themes related to the experience of listening to the lectures and the potential for incidental vocabulary gains from this experience. While at times the student found the experience of listening to the EMI lectures difficult, he still felt it was a rewarding experience as it provided him with an opportunity to prepare for the types of lectures he would encounter during formal university studies. He did, however, mention a few unfounded beliefs regarding lectures given by non-native and native speakers of English. This indicated a need for orientation training for incoming first year students. There was also evidence from the interview data that there is a need for bridging the gap between secondary and tertiary EMI education through teacher in-service training.

While this case study was able to underscore the importance of giving due attention to EMI lectures in the Macau educational context, it was not without its limitations. This case study drew on the data collected from a single course in one subject area of higher education. Without corpus analyses having been conducted on other first year courses in other subject areas, the present results are most applicable to the subject of English language teaching at one higher education institution in Macau. Likewise, only one student was recruited to have his incidental learning assessed with vocabulary tests and have his perceptions of the EMI lectures collected through an interview. In addition, the student only listened to the lectures and did not attend the lectures as is the case in formal course enrollment. It is likely that lectures given in a classroom would be attended by students in a different manner, reducing the ecological validity of the current case study. A future comparison study of students attending in-class and out-of-class lectures could be executed to fully assess the suitability of this method of collected incidental vocabulary acquisition data.

The present study was mainly concerned with the potentials for and the actual incidental acquisition of vocabulary knowledge from listening to EMI lectures. While the use of the three vocabulary assessment measures were able to provide useful information on the student’s acquisition of three aspects of target word knowledge from listening to the lectures, these assessments relied on the use of orthographic vocabulary tests of words encountered through aural input. It would have been more suitable to have administered the vocabulary assessments in an aural format rather than a written format (e.g., Ha, 2021, 2022b); however, this route would have also introduced other potential confounding variables (e.g., pronunciation or accent of the language spoken on the test). As research has found a link between aural single-word knowledge and L2 listening comprehension (Cheng et al., 2022), future research should look carefully into the possibility of adapting and validating existing listening vocabulary levels tests for assessing L1-Chinese learners’ existing English vocabulary knowledge (e.g., McLean et al., 2015; Ha, 2021, Ha, 2022a). In addition, researchers should consider development of novel incidental vocabulary acquisition tests with spoken language that would be considered as intelligible to the targeted population of students. If such routes are taken, accent and pronunciation should be carefully controlled.

We also would like to make it known that we acknowledge the current debates and the criticisms that have been raised in the vocabulary assessment literature on the use of particular vocabulary assessments reliant on the construct of word family (Stewart et al., 2021; Webb, 2021b); however, we also acknowledge that some of these arguments are supported with empirical findings drawn almost entirely from learners with L1-Japanese backgrounds. Evidence through replicated empirical studies is indeed needed to determine for certain whether word form variation from language input can affect vocabulary acquisition (Reynolds, 2015b; Webb, 2021c).

Likewise, we also acknowledge the inherent limitations of multiple choice or matching formats used for data collection with the standardized and novel assessments in the present study. However, teachers and researchers alike must make a balance between practicality and reliability when assessing vocabulary knowledge (Nation, 2013b). This is exactly what we have done in the present study. Thus, we highly suggest an err on caution when criticizing existing tools at the disposal of vocabulary researchers and instead see whether these criticisms can be supported from multiple L2 learner populations (see Snoder and Laufer, 2022 for an interesting study with L1 Swedish learners of L2 English as an example).

As with all studies with a limited sample from a targeted population, this study’s results are likely to have been biased and should not be generalized to the entire first year Macau undergraduate student body. Likewise, only a small number of words were assessed in this study. Although these words were carefully selected based on their properties and the participant’s linguistic background, it is likely that the student could have acquired other vocabulary that did not appear on the assessments. Future studies should address some of these limitations by collecting lectures from multiple courses within the same and different disciplines as well as from different lecturers from different higher education institutions. This could provide more evidence to make more general claims about the Macau higher education context. Likewise, a much larger student sample should be recruited to gather their perceptions regarding higher education EMI lectures and to assess their incidental knowledge of vocabulary of a larger number of items from viewing such lectures. Still, this study does offer valuable insights to future researchers and has shown that this is a valid area of research that deserves further attention.

## Data availability statement

The original contributions presented in this study are included in the article/Supplementary material, further inquiries can be directed to the corresponding author.

## Ethics statement

The studies involving human participants were reviewed and approved by Sub-Panel on Social Science and Humanities Research at the University of Macau. Written informed consent to participate in this study was provided by the participants’ legal guardian/next of kin.

## Author contributions

BR contributed to conceptualization, methodology, data collection, transcription, resources, supervision, project administration, and funding acquisition, charge of review and editing, and designed the vocabulary assessments. XX and QP conducted formal analysis and was validated by BR. All authors wrote the first draft of the manuscript. QP proofed the manuscript. XX and QP were in charge of data curation and software. XX was in charge of language translation. All authors contributed to manuscript revision during the review process, read, and approved the submitted version.

## Footnotes

1 Non-EMI courses include lectures collected in universities in New Zealand, the UK, Ireland, and the U.S (see Dang (2022) for further discussion on non-EMI courses).
