# Supplementary material for: Incidental vocabulary acquisition from listening to English teacher education lectures: A case study from Macau higher education
Source: Front Psychol. 2022 Sep 2;13:993445. doi: 10.3389/fpsyg.2022.993445 (PMC9479006; doi:10.3389/fpsyg.2022.993445)
Supplement: Supplementary file 1 [file Data_Sheet_1.docx]

Supplementary Material

# Supplementary Data

**Cumulative Lexical Coverage Plus Proper Nouns and Marginal Words Across Individual Lectures**

Figures in **bold** indicate 98% coverage.

| Wordlist | lec1 | lec2 | lec3 | lec4 | lec5 | lec6 | lec7 | lec8 | lec9 | lec10 |
| --- | --- | --- | --- | --- | --- | --- | --- | --- | --- | --- |
| 1,000 | 85.92 | 83.3 | 81.34 | 84.8 | 83.57 | 87.79 | 84.97 | 85.6 | 88.33 | 89.67 |
| 2,000 | 94.26 | 90.88 | 90.72 | 92.1 | 92.28 | 94.21 | 92.69 | 94.19 | 95.09 | 96.48 |
| 3,000 | **98.21** | 96.49 | 97.03 | **98.6** | 97.84 | **98.5** | 97.24 | 97.26 | **98.23** | **98.44** |
| 4,000 | 98.32 | 97 | 97.42 | 99.12 | **98.19** | 98.72 | 97.99 | 97.92 | 98.62 | 98.8 |
| 5,000 | 98.47 | **98.21** | **98.76** | 99.36 | 98.48 | 98.72 | **98.22** | **98.21** | 98.77 | 99.05 |
| 6,000 | 98.47 | 98.21 | 99.03 | 99.52 | 98.83 | 98.72 | 98.28 | 98.87 | 98.88 | 99.17 |
| 7,000 | 99.21 | 99.55 | 99.06 | 99.6 | 99.36 | 98.72 | 98.45 | 98.95 | 98.92 | 99.26 |
| 8,000 | 99.25 | 99.68 | 99.18 | 99.6 | 99.48 | 98.72 | 98.45 | 98.95 | 98.96 | 99.32 |
| 9,000 | 99.36 | 99.87 | 99.18 | 99.6 | 99.48 | 98.72 | 98.45 | 98.95 | 98.97 | 99.32 |
| 10,000 | 99.36 | 99.87 | 99.18 | 99.6 | 99.48 | 98.85 | 99.26 | 98.99 | 99.1 | 99.32 |
| 11,000 | 99.36 | 99.87 | 99.21 | 99.64 | 99.48 | 99.04 | 99.32 | 98.99 | 99.1 | 99.32 |
| 12,000 | 99.36 | 99.87 | 99.21 | 99.64 | 99.48 | 99.04 | 99.32 | 98.99 | 99.1 | 99.32 |
| 13,000 | 99.36 | 99.87 | 99.24 | 99.64 | 99.48 | 99.1 | 99.32 | 98.99 | 99.17 | 99.32 |
| 14,000 | 99.36 | 99.87 | 99.24 | 99.68 | 99.48 | 99.13 | 99.32 | 98.99 | 99.17 | 99.32 |
| 15,000 | 99.36 | 99.87 | 99.24 | 99.68 | 99.48 | 99.13 | 99.32 | 98.99 | 99.48 | 99.37 |
| 16,000 | 99.36 | 99.87 | 99.24 | 99.68 | 99.48 | 99.13 | 99.38 | 98.99 | 99.49 | 99.37 |
| 17,000 | 99.36 | 99.87 | 99.39 | 99.68 | 99.48 | 99.13 | 99.38 | 98.99 | 99.49 | 99.37 |
| 18,000 | 99.36 | 99.87 | 99.39 | 99.68 | 99.48 | 99.13 | 99.38 | 98.99 | 99.49 | 99.37 |
| 19,000 | 99.36 | 99.87 | 99.39 | 99.68 | 99.48 | 99.13 | 99.38 | 98.99 | 99.49 | 99.37 |
| 20,000 | 99.36 | 99.87 | 99.39 | 99.68 | 99.48 | 99.13 | 99.38 | 98.99 | 99.5 | 99.37 |
| 21,000 | 99.36 | 99.87 | 99.39 | 99.68 | 99.48 | 99.13 | 99.38 | 98.99 | 99.5 | 99.37 |
| 22,000 | 99.36 | 99.87 | 99.39 | 99.68 | 99.48 | 99.13 | 99.38 | 98.99 | 99.5 | 99.37 |
| 23,000 | 99.36 | 99.87 | 99.39 | 99.68 | 99.48 | 99.13 | 99.38 | 98.99 | 99.53 | 99.37 |
| 24,000 | 99.36 | 99.87 | 99.39 | 99.68 | 99.48 | 99.13 | 99.38 | 98.99 | 99.53 | 99.37 |
| 25,000 | 99.36 | 99.87 | 99.39 | 99.68 | 99.48 | 99.13 | 99.38 | 98.99 | 99.53 | 99.37 |
| proper nouns | 0.74 | 0.13 | 0.39 | 0.44 | 0.35 | 0.5 | 1.27 | 0.46 | 5.52 | 2.28 |
| marginal words | 0 | 0.06 | 0.03 | 0 | 0 | 0.03 | 0 | 0 | 0.1 | 0.13 |
| off-list words | 0 | 0 | 0.09 | 0 | 0.06 | 0 | 0 | 0 | 0.1 | 0.14 |
| number of tokens | 2,686 | 1,569 | 3,360 | 2,493 | 1,710 | 3,194 | 1,736 | 2,409 | 7,126 | 8,518 |

| Wordlist | lec11 | lec12 | lec13 | lec14 | lec15 | lec16 | lec17 | lec18 | lec19 | lec20 |
| --- | --- | --- | --- | --- | --- | --- | --- | --- | --- | --- |
| 1,000 | 89.98 | 88.46 | 89.66 | 89.37 | 89.26 | 87.75 | 86.45 | 87.33 | 84.28 | 83.46 |
| 2,000 | 95.97 | 94.4 | 94.25 | 95.01 | 94.71 | 93.58 | 92.27 | 93 | 92.81 | 91.62 |
| 3,000 | **98.32** | **99.31** | **98.21** | 97.93 | **98.34** | 97.03 | 96.17 | 97.01 | 97.35 | 97.62 |
| 4,000 | 98.75 | 99.51 | 98.5 | **98.28** | 98.54 | 97.81 | 96.48 | 97.37 | 97.78 | **98.12** |
| 5,000 | 98.9 | 99.71 | 98.63 | 98.48 | 98.8 | **98.17** | 96.82 | **98.52** | **98.8** | 99.18 |
| 6,000 | 99.08 | 99.85 | 98.84 | 98.63 | 99.26 | 98.25 | **98.77** | 98.76 | 99.05 | 99.41 |
| 7,000 | 99.18 | 99.85 | 98.92 | 98.81 | 99.49 | 98.95 | 98.85 | 98.94 | 99.19 | 99.53 |
| 8,000 | 99.26 | 99.85 | 98.96 | 98.89 | 99.52 | 98.97 | 98.88 | 99.03 | 99.19 | 99.55 |
| 9,000 | 99.27 | 99.85 | 98.96 | 98.91 | 99.52 | 98.97 | 98.91 | 99.03 | 99.19 | 99.55 |
| 10,000 | 99.27 | 99.92 | 99 | 98.91 | 99.55 | 99 | 98.94 | 99.06 | 99.21 | 99.55 |
| 11,000 | 99.27 | 99.92 | 99 | 99.05 | 99.55 | 99.02 | 98.94 | 99.06 | 99.23 | 99.57 |
| 12,000 | 99.3 | 99.92 | 99.08 | 99.11 | 99.58 | 99.02 | 98.94 | 99.06 | 99.28 | 99.57 |
| 13,000 | 99.3 | 99.92 | 99.08 | 99.11 | 99.58 | 99.02 | 98.97 | 99.06 | 99.28 | 99.61 |
| 14,000 | 99.34 | 99.92 | 99.08 | 99.11 | 99.58 | 99.02 | 98.97 | 99.06 | 99.28 | 99.61 |
| 15,000 | 99.37 | 99.92 | 99.08 | 99.16 | 99.65 | 99.02 | 98.97 | 99.42 | 99.31 | 99.61 |
| 16,000 | 99.38 | 99.92 | 99.08 | 99.16 | 99.65 | 99.02 | 99.05 | 99.42 | 99.31 | 99.61 |
| 17,000 | 99.38 | 99.92 | 99.08 | 99.16 | 99.65 | 99.02 | 99.05 | 99.45 | 99.39 | 99.68 |
| 18,000 | 99.39 | 99.92 | 99.12 | 99.16 | 99.65 | 99.02 | 99.05 | 99.45 | 99.39 | 99.68 |
| 19,000 | 99.39 | 99.92 | 99.12 | 99.16 | 99.65 | 99.02 | 99.05 | 99.45 | 99.39 | 99.68 |
| 20,000 | 99.39 | 99.92 | 99.12 | 99.16 | 99.65 | 99.02 | 99.05 | 99.45 | 99.39 | 99.68 |
| 21,000 | 99.39 | 99.92 | 99.12 | 99.16 | 99.65 | 99.02 | 99.05 | 99.45 | 99.39 | 99.68 |
| 22,000 | 99.39 | 99.92 | 99.12 | 99.16 | 99.65 | 99.02 | 99.05 | 99.45 | 99.39 | 99.68 |
| 23,000 | 99.39 | 99.92 | 99.12 | 99.16 | 99.65 | 99.02 | 99.05 | 99.45 | 99.39 | 99.68 |
| 24,000 | 99.39 | 99.92 | 99.12 | 99.16 | 99.65 | 99.02 | 99.05 | 99.45 | 99.39 | 99.68 |
| 25,000 | 99.39 | 99.92 | 99.12 | 99.16 | 99.65 | 99.02 | 99.05 | 99.45 | 99.39 | 99.68 |
| proper nouns | 1.63 | 1.64 | 1.09 | 2.92 | 4.18 | 1.7 | 0.93 | 2.59 | 0.32 | 0.14 |
| marginal words | 0.11 | 0.27 | 0.21 | 0.12 | 0.1 | 0.13 | 0.06 | 0.18 | 0.02 | 0.02 |
| off-list words | 0.08 | 0 | 0.08 | 0.03 | 0 | 0.02 | 0.11 | 0.21 | 0.03 | 0.04 |
| number of tokens | 7,985 | 1,465 | 2,396 | 6,547 | 3,062 | 6,172 | 3,542 | 3,317 | 6,297 | 5,652 |

| Wordlist | lec21 | lec22 | lec23 | lec24 | lec25 | lec26 | lec27 |
| --- | --- | --- | --- | --- | --- | --- | --- |
| 1,000 | 83.45 | 81.16 | 86.4 | 85.84 | 87.7 | 88.28 | 86.04 |
| 2,000 | 90.39 | 89.96 | 93.1 | 92.41 | 93.71 | 93.52 | 92.3 |
| 3,000 | 94.94 | 95.44 | 96.93 | 97.99 | 97.18 | 96.81 | 96.87 |
| 4,000 | 95.41 | 95.93 | **98.14** | **98.53** | 97.72 | 97.51 | 97.55 |
| 5,000 | 95.79 | 96.18 | 99.07 | 98.72 | **98.99** | **98.41** | **98.64** |
| 6,000 | 95.82 | 96.3 | 99.17 | 98.88 | 99.31 | 99.06 | 99.22 |
| 7,000 | 96.04 | 96.85 | 99.5 | 99.02 | 99.39 | 99.21 | 99.4 |
| 8,000 | 96.04 | 96.85 | 99.56 | 99.05 | 99.62 | 99.4 | 99.59 |
| 9,000 | 96.04 | 96.97 | 99.57 | 99.11 | 99.62 | 99.4 | 99.6 |
| 10,000 | 96.2 | 97.46 | 99.58 | 99.14 | 99.62 | 99.42 | 99.61 |
| 11,000 | 96.2 | 97.46 | 99.59 | 99.15 | 99.65 | 99.51 | 99.62 |
| 12,000 | 96.2 | 97.46 | 99.59 | 99.15 | 99.65 | 99.53 | 99.62 |
| 13,000 | 96.2 | 97.58 | 99.62 | 99.15 | 99.67 | 99.53 | 99.65 |
| 14,000 | 96.2 | 97.58 | 99.63 | 99.15 | 99.67 | 99.68 | 99.68 |
| 15,000 | 96.2 | 97.58 | 99.63 | 99.15 | 99.67 | 99.7 | 99.7 |
| 16,000 | 96.2 | 97.58 | 99.63 | 99.15 | 99.69 | 99.7 | 99.7 |
| 17,000 | 96.29 | 97.76 | 99.72 | 99.18 | 99.7 | 99.7 | 99.74 |
| 18,000 | 96.29 | 97.76 | 99.72 | 99.18 | 99.7 | 99.7 | 99.74 |
| 19,000 | 96.29 | 97.76 | 99.72 | 99.18 | 99.7 | 99.7 | 99.74 |
| 20,000 | 96.29 | 97.76 | 99.73 | 99.18 | 99.71 | 99.7 | 99.75 |
| 21,000 | 96.29 | 97.76 | 99.73 | 99.18 | 99.71 | 99.7 | 99.75 |
| 22,000 | 96.29 | 97.76 | 99.73 | 99.18 | 99.71 | 99.7 | 99.75 |
| 23,000 | 96.29 | 97.76 | 99.73 | 99.18 | 99.71 | 99.7 | 99.75 |
| 24,000 | 96.29 | 97.76 | 99.73 | 99.18 | 99.71 | 99.7 | 99.75 |
| 25,000 | 96.29 | 97.76 | 99.73 | 99.18 | 99.71 | 99.71 | 99.75 |
| proper nouns | 0.22 | 0.18 | 0.17 | 0.27 | 0.39 | 0.69 | 0.17 |
| marginal words | 0 | 0 | 0.01 | 0.1 | 0.06 | 0.07 | 0.03 |
| off-list words | 0.03 | 0.06 | 0.05 | 0.37 | 0.06 | 0.04 | 0.02 |
| number of tokens | 3,185 | 1,625 | 9,672 | 6,987 | 8,691 | 8,108 | 8,994 |

# Supplementary Instruments

**Receptive Form and Productive Meaning Test**

Directions: For each item, please select one among the four that is spelled correctly. Then in the space provided on the right, provide a Chinese translation of the word’s meaning.

| No. | Item | Translation |
| --- | --- | --- |
| 1 | ingrecant  inglecant  increment  inwrecant |  |
| 2 | nulafy  nangacy  fastary  fallacy |  |
| 3 | nertatize  nertatism  normative  nalmatize |  |
| 4 | cognate  codgate  coxhate  coxgate |  |
| 5 | limistic  fetistic  fesistic  holistic |  |
| 6 | rerost  recast  regats  relals |  |
| 7 | scaffold  claffoth  draffoct  draffoin |  |
| 8 | litixal  lexical  fesixal  fosical |  |
| 9 | podient  salient  rosient  dosient |  |
| 10 | syntik  syntax  syndex  syntow |  |

**Receptive Meaning Test**

Directions: Please select the meaning of the word. If you do not know the meaning of this word, please select option e “我不知道.”

| 1 | cognate | a) 與另一個詞有不同的拼寫或含義  b) 與另一個詞有相同的拼寫或含義  c) 與另一個詞同源  d) 與另一個詞不同源  e) 我不知道 |
| --- | --- | --- |
| 2 | recast | a) 以更正的形式向學習者重複錯誤  b) 使用升調以更正的形式向學習者重複一個錯誤  c) 以未更正的形式向學習者重複錯誤  d) 使用降調以更正的形式向學習者重複一個錯誤  e) 我不知道 |
| 3 | lexical | a) 與一種語言的拼寫相關  b) 與一種語言的詞匯相關  c) 與一種語言的發音相關  d) 與一種語言的語法相關  e) 我不知道 |
| 4 | fallacy | a) 一個正確的想法，但許多人認為是錯誤的  b) 一個錯誤的想法，但許多人認為是正確  c) 一個正確想法，且許多人認為是正確的  d) 一個錯誤想法，且許多人認為是錯誤的  e) 我不知道 |
| 5 | syntax | a) 在一種語言中，將字母組成書面詞彙的方式  b) 在一種語言中，使用詞彙和短語造句的方式  c) 在一種語言中，將音素組成口語詞彙的方式  d) 在一種語言中，使用句子和發音組成段落的方式  e) 我不知道 |
| 6 | scaffold | a) 在整個學習過程中， 只有一位老師給予一位學生的支持  b) 在整個學習過程中，同學或老師未給予一位學生的支持  c) 在整個學習過程中，給予一位學生的支持  d) 在整個學習過程中，只有一位同學給予一位學生的支持  e) 我不知道 |
| 7 | normative | a) 用來澄清或解釋某件事  b) 用來描述或闡明某件事  c) 無視或違反行為標準或規則  d) 描述或設定行為標準或規則  e) 我不知道 |
| 8 | salient | a) 容易注意到  b) 不容易注意到  c) 不容易改變  d) 容易改變  e) 我不知道 |
| 9 | increment | a) 數字或數量的減少  b) 使某些東西變大或數量變多  c) 使某些東西變小或數量變少  d) 數字或數量的增加  e) 我不知道 |
| 10 | holistic | a) 渴望成功的  b) 考慮整體  c) 考慮局部  d) 不渴望成功的  e) 我不知道 |

**Semi-structured Interview Questions**

Please briefly introduce yourself.

What is your perception of the vocabulary used in the lectures?

What were the difficulties you encountered when listening to the lectures?

How well did your (vocabulary) learning in secondary school prepare you for listening to the lectures?

What are the words that you picked up (learned) from these lectures?

Which words did you find difficult to learn from these lectures?

What did you do when you encountered unknown words when listening to the lectures?

Which lecture helped you to learn the most new words?

Which lecture helped you to learn the fewest new words?

How do you plan to use the vocabulary learned from these lectures in your future study or life?

From your perspective, what could have helped you learn more vocabulary from listening to the lectures?

On a scale from 0 to 6, how do you rate the experience of listening to these lectures?

How important to you was the experience of listening to these EMI lectures?

What advice would you give to future teachers that deliver EMI lectures for the same course?

What advice would you give to your secondary school teachers that could prepare you to learn content from listening to EMI lectures like the ones you listened to?

Please provide any additional comments or ask any questions you may have.
